# Synergistic Effects of Gold Nanocages in Hyperthermia and Radiotherapy Treatment

**DOI:** 10.1186/s11671-016-1501-y

**Published:** 2016-06-02

**Authors:** Ai-wei Zhang, Wei-hua Guo, Ya-fei Qi, Jian-zhen Wang, Xiang-xing Ma, De-xin Yu

**Affiliations:** Department of Radiology, Qilu Hospital, Shandong University, Jinan, 250012 People’s Republic of China; Key Laboratory of Colloid and Interface Chemistry, Ministry of Education, Department of Chemistry, Shandong University, Jinan, People’s Republic of China; Department of Radiotherapy, Qilu Hospital, Shandong University, Jinan, People’s Republic of China

**Keywords:** Gold nanocages (GNCs), Hyperthermia, Radiosensitivity, Breast cancer, Targeted cancer therapy

## Abstract

Gold nanocages (GNCs) are a promising material that not only converts near infrared (NIR) light to heat for the ablation of tumors but also acts as a radiosensitizer. The combination of hyperthermia and radiotherapy has a synergistic effect that can lead to significant tumor cell necrosis. In the current study, we synthesized GNCs that offered the combined effects of hyperthermia and radiotherapy. This combination strategy resulted in increased tumor cell apoptosis and significant tumor tissue necrosis. We propose that GNCs can be used for clinical treatment and to potentially overcome resistance to radiotherapy by clearly increasing the antitumor effect.

## Background

According to statistics reported in 2014, breast cancer is one of the most commonly diagnosed cancers and is a leading cause of cancer-related mortality among women [1] Since 2008, the incidence and mortality of breast cancer have increased by 23 and 14 %, respectively, in females worldwide [2]. Although many advances have been made in the early treatment of breast cancer, triple-negative breast cancer (TNBC), which lacks the expression of estrogen receptor, progesterone receptor, and human epidermal growth factor receptor 2 (Her2) [3, 4], is still an obstacle for researchers. Non-selective targeted therapy may lead to tumor recurrence and significant side effects [5]. To address these problems, nanotechnology, which is minimally invasive, has been introduced in breast cancer for simultaneous imaging and theranostics [6, 7]. The application of nanotechnology to cancer treatment typically involves the use of nanomaterials that can be conjugated to biomolecules, such as antibodies, peptides, drugs, and imaging reporters, to increase therapeutic efficacy [4, 8–12]. Photothermal therapy (PTT), a minimally invasive therapy, utilizes nanoparticle-mediated conversion of light into heat, which can be used to increase local temperatures [13–15]. Because of the enhanced permeability and retention (EPR) effect [8, 16] and receptor-mediated endocytosis (RME), nanoparticles conjugated to specific antibodies can easily permeate leaky tumor blood vessels and selectively target cancer cells [17, 18]. Numerous studies have demonstrated that temperatures over 40 °C [13] can alter a tumor’s microenvironment and signal transduction properties [19, 20], causing irreversible damage to proteins and resulting in DNA double-strand repair deficiency [11, 19]. As such, PTT represents an improved form of cancer treatment. Many nanomaterials, including gold nanoparticles (AuNPs) [7, 19], carbon nanotubes (CNTs) [21], iron oxide nanoparticles [22], and grapheme oxides (GOs) [23], have been developed for use in PTT. However, when the absorption band of a nanoparticle falls within ultraviolet (UV) and visible light spectra, PTT becomes infeasible in clinical practice because UV and visible light show poor penetration into human tissues [19]. The optimal wavelength for human tissue penetration ranges between 700 and 1000 nm (e.g., near infrared, NIR) [13, 19]. In this wavelength range, there is an important biological transparency window in which water molecules exhibit low absorption and scattering of light; thus, light at these wavelengths can effectively penetrate deep into tissue [7]. The light transmittance of NIR wavelengths is approximately 10 cm, and for most breast cancers, this is sufficient for PTT. Owing to their large optical absorption cross-sections, gold nanocages (GNCs), which absorb light within the NIR spectrum [7], provide excellent optical-to-thermal energy conversion efficiency compared to other gold nanomaterial geometries, such as AuNPs or nanorods [24]. GNCs possess a novel nanostructure that is small in size, is easy to synthesize, possesses wavelength selectivity, exhibits low cytotoxicity, and has excellent biocompatibility [25–29]. Therefore, GNCs are excellent photothermal transducers and have been widely used in translational research.

However, it remains challenging to fully eradicate cancer cells using PTT due to the limited penetration of NIR, especially for deep tumors, making this approach difficult to implement in clinical treatment [19]. Many studies have shown that the application of heat can improve the therapeutic effects of radiotherapy (RT) or chemotherapy [30, 31]. RT in particular is extremely important to oncology due to its ability to kill tumor cells [16, 19]. Indeed, over 50 % of patients with cancer receive curative or palliative RT [32, 33]. Nevertheless, as a single modality, it is difficult to control the dose of ionizing radiation in RT to eradicate all cancer cells due to the radioresistance of tumor cells and the need to avoid applying RT to normal tissue [32–34]. Two strategies have been implemented to overcome this challenge: (1) the use of radioprotectors, such as amifostine [35] and CBLB502 [36], which can reduce the harm of ionizing radiation to normal cells; (2) the use of radiosensitizers, which can enhance the delivery of ionizing radiation to tumor tissue while causing little damage to normal cells [37]. Conventional radiosensitizers, such as misonidazole, are not capable of targeting tumor tissues [33]. Materials with high atomic numbers (*Z*), such as gold, silver, iodine, and gadolinium, have received increasing attention for their excellent radiosensitization effects. Many studies have shown that the effects of RT are closely linked to the atomic radii of radioprotectors [16]. Santos et al. found that the use of iodine (*Z* = 53) contrast media in combination with X-rays can achieve stronger inhibition of tumor growth compared to the use of X-rays alone [38]. However, iodine is rapidly cleared from circulation, which is not beneficial for treatment [16, 39]. Gadolinium (*Z* = 64) and gold (*Z* = 79) have also been used to enhance the effects of RT. Gold is considered an excellent radiosensitizer due to its high atomic number and easy modifiability, which can be used to increase the dose of radiation delivered to a tumor site, reducing side effects in surrounding normal tissue [33, 40–42]. The mass attenuation coefficient of gold is 2.7-fold higher than that of iodine [43] and approximately 1000-fold higher than that of any soft tissue [44]. Many studies have shown that there is prominent photoelectric absorption in the kilovoltage (kV) energy range when using gold. However, because of the limited penetration to soft tissue, using a combination of KV X-rays and GNCs could only be limited to superficial tumors. Megavoltage (MV) X-rays are essential for clinical radical RT both for the treatment of superficial tumors and to achieve adequate dosing of deep tumors [40, 41, 45]. Jain et al. applied GNPs to MDA-MB-231 cells to determine the enhancement effect following irradiation with 160 kVp, 6 MV, and 15 MV X-rays, which produced radiation sensitizer enhancement ratios (SERs) of 1.41,1.29, and 1.16, respectively [46]. Therefore, GNCs serve as an excellent nanomaterial choice not only for their radiosensitizer properties, which increase radiation absorption at tumor sites [19], but also for use in PTT.

Several studies have shown that hyperthermia is one of the most effective radiosensitizers [19]. PTT can increase the temperature of a tumor, which can lead to tumor oxygenation, thus sensitizing the tumor to X-ray radiation [47]. In addition, PTT can also enhance the effects of RT by attenuating the repair of double-strand breaks (DSBs) caused by RT [7, 48]. In summary, co-treatment with PPT and RT can reduce the dose of antitumor irradiation needed while simultaneously providing enhanced therapeutic effects [41].

In the current study, we evaluated the synergistic effects of GNCs in PTT and RT both in vitro and in vivo. This nanomaterial has the potential to be used in cancer theranostics. To improve the nanomaterial’s biocompatibility, SH-PEG-COOH was used to coat the GNCs. Furthermore, the GNCs underwent bio-conjugation to anti-CD44 monoclonal antibodies to enhance their targeting of cancer cells. We compared naked GNCs, PEG-GNCs, and CD44–PEG-GNCs in 4T1 cells and analyzed the associated cytotoxicity after 24 and 48 h. Double Hoechst/PI staining, cell apoptosis assays, and clonogenic survival assays were used to evaluate the effects of the co-treatment. Subsequently, the synergistic effects of the combination were studied in vivo by assessing tumor necrosis after the delivery of 4 Gy of irradiation. We demonstrated that our nanosystem can selectively target breast cancer cells and that the co-application of PTT and RT produces synergistic effects.

## Methods

### Reagents and Materials

Silver nitrate (AgNO_3_), gold (III) chloride trihydrate (HAuCl_4_ · 3H_2_O), trisodium citrate dehydrate (Na_3_Ct · 2H_2_O), N-hydroxy succinimide (NHS), and 1-ethyl-3-(3-dimethyl aminopropyl) carbodiimide (EDC) were obtained from Sigma-Aldrich, USA. SH-PEG-COOH was purchased from Shanghai Xibao Medpep Co. The Cell Counting Kit-8 assay (CCK-8) and an apoptosis detection kit were obtained from BestBio Co. Hoechst/PI was purchased from Solarbio Science & Technology Co. 4T1 cells were available from the cell collection of the China Center for Type Culture Collection. Other cell culture reagents were obtained from Gibco. Ltd. Phosphate-buffered solution (PBS) (pH 7.4, 0.1 M) was prepared with 0.1 M Na_2_HPO_4_, 0.1 M KH_2_PO_4_, and 0.1 M KCl and was used as the working buffer solution.

### Cell Culture

4T1 cells were cultured in Roswell Park Memorial Institute medium (RPMI-1640) supplemented with 10 % (*v*/*v*) fetal bovine serum at 37 °C with 5 % CO_2_. Cells in logarithmic growth phase were used for the experiments.

### Synthesis of Gold Nanocages (GNCs)

For the synthesis of GNCs, a silver colloid was first prepared. Silver nitrate (180 mg) was added to Milli-Q water (100 ml) under stirring conditions. The solution was heated to 100 °C, followed by the addition of trisodium citrate dihydrate (1 ml, 1 *w*/*w*). The solution was boiled for 30 min and then cooled to room temperature. Via the galvanic replacement reaction, the silver colloid was converted into GNCs under refluxing conditions [11, 49]. First, the prepared fresh silver colloid (10 ml) was heated to 60 °C under magnetic stirring, and then a HAuCl_4_ solution (5 ml, 1 mmol/l) was added at a rate of 45 ml/h with a syringe pump. The solution was heated for 30 min and then cooled to room temperature. Then, the solution was concentrated at 8000 rpm for 10 min. The size and morphology of the GNCs were characterized using transmission electron microscopy (TEM) and UV–vis spectroscopy.

### Synthesis of CD44 Antibody-Conjugated PEG-Modified GNCs (GNC-PEG-CD44)

PEG was used as a linker to conjugate the CD44 antibody to the GNCs. First, an aqueous PEG solution (500 μl, 2 mmol/l) was added to the GNCs (1 ml, 5 nmol/l) while being maintained at 4 °C. Four hours later, the PEGylated GNCs were collected by centrifugation at 8000 rpm for 15 min and were washed 2 times with Milli-Q water to remove the unreacted PEG. Afterward, EDC (10 μl, 40 mg/ml) and NHS (10 μl, 110 mg/ml) were added to activate the carboxyl terminal of PEG at room temperature for 1 h. The mixture was centrifuged and washed with Milli-Q water 2 times to remove the unbound EDC and NHS. Then, monoclonal anti-CD44 antibody (Abcam, ab119863) (5 μl, 1 mg/ml) was added and allowed to react overnight at 4 °C. The conjugated solutions were washed with Milli-Q water, and excess antibody was removed by centrifugation. The obtained immuno GNC samples were resuspended in PBS and sterilized using a 0.2-mm filter for further characterization and application.

### Cellular Uptake Experiment

4T1 cells were cultured in a T25 culture flask and incubated overnight at 37 °C with 5 % CO_2_. Prior to the uptake experiment, the cells were rinsed with PBS (pH 7.4) and incubated with a mixture solution containing culture medium (2 ml) and the immuno GNCs (1 ml, 5 nmol/l). After 24 h, the cells were washed with PBS and collected for TEM observation.

A subset of cells was cultured in a 6-well plate (5 × 10^5^ cells per well) and incubated overnight at 37 °C under 5 % CO_2_. After rinsing twice with PBS, the cells were incubated with immuno GNCs or PEGylated GNCs for 2, 4, 8, 18, and 24 h. At different time points, the cells were collected and digested with aqua regia. Then, inductively coupled plasma mass spectrometry (ICP-MS) was employed to measure the amount of Au using the following formula: *M* (the weight of Au measured by ICP-MS)/[M (the weight of the cells) + *M* (the uptake of GNCs)].

### Cytotoxicity of the GNCs

The cytotoxicity of the GNCs when applied to 4T1 cells was evaluated using a CCK-8 assay. 4T1 cells were seeded into 96-well plates at a density of 5000 cells/well in 100 μl of medium and incubated for 24 h at 37 °C with 5 % CO_2_. Then, media with various concentrations of GNCs (0, 1, 2, or 3 nmol/l) were used to replace the initial medium. After 24 or 48 h, 10 μl of CCK-8 solution was added to each well and incubated for an additional 2 h at 37 °C. The absorbance was measured at 450 nm using a micro-plate reader (Bio-Rad Model 680, Richmond, CA, USA). This experiment was repeated 3 times.

### Photothermal Effects of the GNCs

Aliquots (1.5 mL, 3 nmol/l) of naked GNCs, PEGylated GNCs, or immuno GNCs were added to cuvettes and irradiated under an 808-nm NIR laser (Changchun New Industries Optoelectronics Technology Co., Ltd.) at 2.5 W/cm^2^. During irradiation, the temperatures of the solutions were measured over a 16-min time period using an infrared thermometer (Heng Odd Inc., China). Milli-Q water was used as a control.

### In Vitro Photothermal Therapy

4T1 cells were seeded on 96-well plates at a density of 5000 cells/well in 100 μl of medium and incubated overnight at 37 °C with 5 % CO_2_. The cells were then incubated with 3 nmol/l PEGylated GNCs or immuno GNCs for 24 h. Then, the cells were washed with PBS 2 times to remove the excess GNCs, and fresh culture medium was added to each well. Subsequently, 5 W/cm^2^ or 2.5 W/cm^2^ of 808-nm NIR laser irradiation was applied to the 4T1 cells for 5 min [11, 16]. After NIR light exposure, the cells were maintained at 37 °C under 5 % CO_2_ for 3 h. Finally, a CCK-8 assay was utilized to measure cell viability. This experiment was repeated 3 times.

### Clonogenic Survival Assay

4T1 cells were seeded on 6-well plates at a density of 400 cells/well in 2 ml of medium and incubated overnight. Then, 3 nmol/l PEGylated GNCs or immuno GNCs was added to the medium and incubated for 24 h. The cells were washed 2 times with PBS after incubation and exposed to 2, 4, 6, and 8 Gy of 6-MV X-ray radiation. Then, the cells were washed twice with PBS and incubated in fresh culture medium for 9–14 days to form colonies. The colonies were fixed in 4 % paraformaldehyde and stained with 0.1 % crystal violet. Colonies containing 50 cells were counted. The plating efficiency was calculated as (number of colonies)/(number of inoculated cells as a control), and the surviving fraction was defined as (number of colonies)/(number of inoculated cells) × plating efficiency.

### Double Hoechst/PI Staining and Cell Apoptosis Analysis

4T1 cells (3 × 10^5^) were incubated with 3 nM PEGylated GNCs or immuno GNCs for 24 h and then irradiated either with NIR at a power density of 2.5 W/cm^2^ or with X-rays at a dose of 4 Gy. The cells were collected after 3 h and washed twice with cold PBS. Hoechst/PI dye was then added and reacted at 4 °C. After 15 min, PBS was used to terminate the reaction. The results were observed under a fluorescence microscope (Olympus IX81). Additionally, an apoptosis detection kit was used in tandem with flow cytometry (Becton–Dickinson, CA, USA) to detect the number of apoptotic cells. To accomplish this, after treatment, the cells were resuspended in 400-μl Annexin V-FITC binding buffer and stained via the sequential addition of Annexin V-FITC (5 μl) and PI (10 μl) for visualization. This experiment was repeated 3 times.

### Mice and Tumors

Four-week-old female BALB/c mice were purchased from Jinan PengYue Laboratory Animal Co., Ltd (China). All animal procedures were performed in accordance with institutional animal use and care regulations. The mice were subcutaneously implanted with 4T1 cells in their right rear flank area. A total of 5 × 10^6^ cells in 1 ml of RPMI-1640 medium were used for implantation. After the tumor reached approximately 6–8 mm in diameter, the animals were anesthetized with 0.1 ml of 10 % chloral hydrate and subjected to treatment.

### In Vivo Photothermal Therapy and Radiotherapy

To evaluate therapeutic effects in vivo, the mice were randomized into several groups (*n* = 3 per group): (1) a control group, (2) a PBS intravenous (i.v.) injection + PTT group, (3) a PBS i.v. injection + RT group, (4) a PBS i.v. injection + PTT + RT group, (5) a PEG-GNC i.v. injection + PTT group, (6) a PEG-GNC i.v. injection + RT group, (7) a CD-PEG-GNC i.v. injection + PTT group, (8) a CD-PEG-GNC i.v. injection + RT group, (9) a CD-PEG-GNCs i.v. injection + PTT + RT group, (10) a GNC intratumoral (i.t.) injection + PTT group, (11) a GNC i.t. injection + RT group, and (12) a GNC i.t. injection + PTT + RT group. For the i.t. injections, we used a syringe pump to deliver 100 μl of 1.5 mg Au/ml GNCs into the center of each tumor mass. The rate of injection was 10 μl/min. PTT and RT were implemented 24 h later. In the control group, PBS was injected into the tumor. For the i.v. injections, 100 μl of 1.5 mg Au/ml of immuno GNCs or PEGylated GNCs was injected into the tail vein. PTT was performed using an NIR laser to irradiate the tumor at 2.5 W/cm^2^ for 5 min with a wavelength of 808 nm. After heating, the mice were exposed to 4 Gy of 6-MV X-ray radiation.

### Hematoxylin and Eosin Staining Assay

All of the tumors were removed from tumor-bearing mice at 3 days after therapy, fixed in 10 % formalin, and stained for H&E analysis.

### Statistical Analysis

SPSS version 13.0 (SPSS Inc., Chicago, IL, USA) was used to perform all statistical analysis. Experimental data were expressed as the mean ± standard deviation (*x* ± *s*). ANOVA and chi-square tests were used to compare the differences between various groups. A *P* value <0.05 was considered statistically significant.

## Results

### Synthesis and Characterization of GNCs

**Fig. 1:**
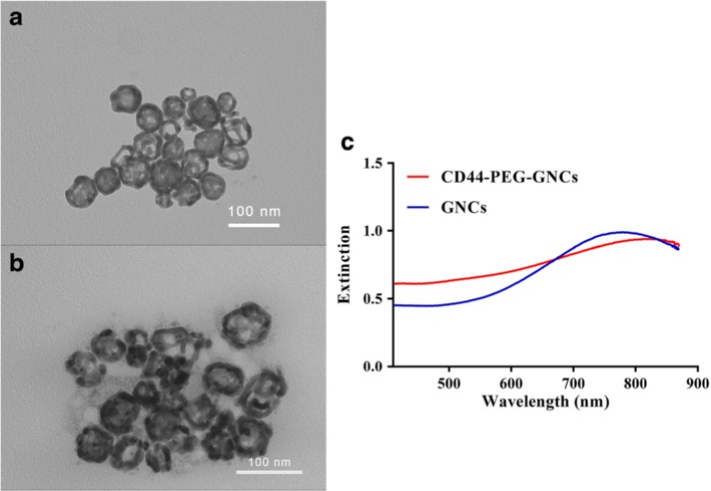
**a** TEM image of GNCs. **b** TEM image of CD44-PEG-GNCs. **c** UV–vis extinction of GNCs and CD44-PEG-GNCs

According to TEM image of GNCs and CD44-PEG-GNCs, as shown in Fig. 1, the mean diameter of our GNCs was 58.14 ± 4 nm, and the wall thickness was 9 ± 1.86 nm. Figure 1a clearly shows the hollow interior and thin shell of the GNCs. A UV–vis extinction spectrum showed that the plasma resonance peak for the GNCs was approximately 780 nm (Fig. 1c), while the peak for the immuno GNCs occurred at approximately 813 nm. A characteristic red shift (≈33 nm) was observed in the localized plasma resonance peak, which resulted from the increased hydrodynamic diameter produced from antibody conjugation. With the increase in the size of the GNCs, the maximum extinction efficiency increased, and the maximum extinction position became red shifted [11, 16, 50–52]. Zeta-potential measurements indicated that both GNCs and CD44-PEG-GNCs were negatively charged (Table 1) and relatively stable [53]. Moreover, GNCs were more negatively charged than after conjugation with antibody. This is likely because GNC conjugation with antibody are in fact a displacement by them of many negatively charged citrate ions, which results in reorganization of the surface charge of the particles and the corresponding change in zeta-potential [54].

**Table 1:** Z-Potentials of GNCs PEG-GNCs and CD44-PEG-GNCs

Nanoparticles	Z-Potentials (mV)
GNCs	−51 ± 5
PEG-GNCs	−36 ± 3
CD44-PEG-GNCs	−34 ± 5

### Expression of CD44 Receptors in 4T1 Cells and Cellular Uptake Efficiency of the GNCs

**Fig. 2:**
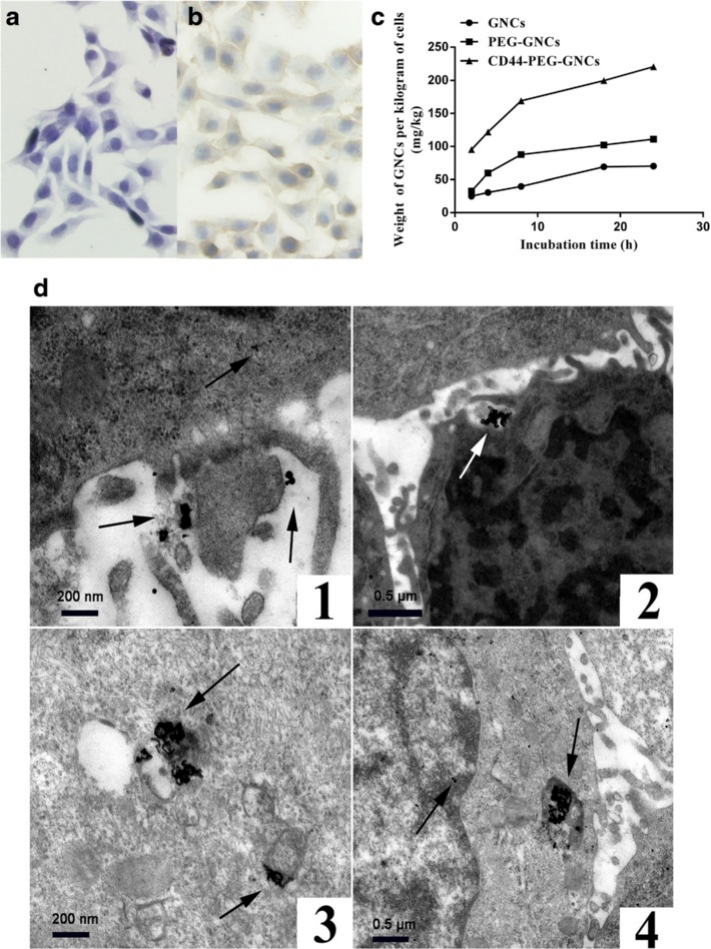
Immunocytochemistry and cellular uptake of the GNCs. **a** Immunocytochemistry for the negative control group of 4T1 cells. **b** Selective immunohistochemical staining of CD44 within the 4T1 cells. **c** Uptake of GNCs, PEG-GNCs versus CD44-PEG-GNCs per kilogram of 4T1 cells, as measured by ICP-MS. **d** (*1*–*2*) TEM images of 4T1 cells incubated with CD44-PEG-GNCs, and the CD44-PEG-GNCs entered the cells through receptor-mediated endocytosis. **d** (*3*) CD44-PEG-GNCs readily aggregated around the organelle membranes. **d** (*4*) CD44-PEG-GNCs aggregated in the organelles

To confirm the targeting ability of the immuno GNCs, we first verified the expression of CD44 receptors on 4T1 cells by immunohistochemistry and then quantified the number of GNCs taken up by 4T1 cells. Figure 2b shows that a large number of CD44 receptors were expressed on the 4T1 cell membrane, while few receptors were expressed in the cytoplasm. The expression of CD44 receptors is a prerequisite for the preparation of CD44-PEG-GNCs. ICP-MS was employed to verify the targeting ability of CD44-PEG-GNCs to 4T1 cells. As shown in Fig. 2c, for the GNCs, PEGylated GNCs, and immuno GNCs, the number of Au taken up by the 4T1 cells was positively related to the incubation time for the first 24 h. Within the first 8 h, the amount of Au taken up by the cells was increasing dramatically, then the rise slightly flat. Moreover, the amount of Au taken up for the group of immuno GNCs was much greater than that for the GNCs and PEGylated GNCs (*P* < 0.05). This result indicates that the CD44-PEG-GNCs show excellent targeting capability to 4T1 cells. To more clearly observe 4T1 cell phagocytosis of immuno GNCs, TEM images were taken after 4T1 cells were incubated with immuno GNCs for 24 h. As shown in Fig. 2d, the GNCs initially aggregated around the cell membranes and then entered the cells via endocytosis. Subsequently, some GNCs were dispersed in the cytoplasm or localized around the organelle membranes while some GNCs entered the organelles.

### Cytotoxicity of GNCs

**Fig. 3:**
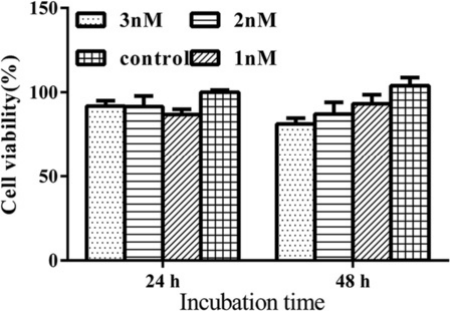
4T1 cells incubated with different concentrations of GNCs (1, 2, and 3 nmol/l) at 24 and 48 h, measured by a CCK-8 assay

Cytotoxicity is an important parameter in assessing whether a nanomaterial can be used for in vivo experiments. After different concentrations of GNCs (0, 1, 2, and 3 nM) were incubated with 4T1 cells, the cytotoxicity of GNCs was measured by CCK-8 assay. Figure 3 indicates that over 48 h, low cytotoxicity was observed for naked GNCs in the concentration range of 1–3 nM in comparison to the control (*P* > 0.05). The cell viability for groups with 3 nmol/l naked GNCs was 91.87 % at 24 h and 81.2 % at 48 h, indicating that the GNCs do not exert any obvious cytotoxicity within this concentration range. Therefore, in the following experiment, we believe that the use of GNCs within 3 nmol/l is safe and feasible.

### Photothermal Effect of GNCs

**Fig. 4:**
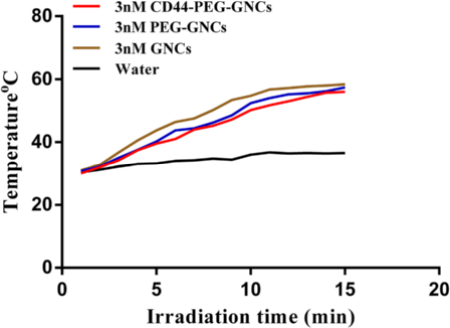
Temperature changes for GNCs, PEG-GNCs, and CD44-PEG-GNCs irradiated by NIR laser

Because of surface plasmon resonance, GNCs can absorb NIR light and convert it into heat. To investigate the photothermal effects of GNCs, we used an NIR laser (808 nm) at a power density of 2.5 W/cm^2^ to irradiate different types of GNCs. We found that during the first 9–10 min of heating, the temperatures of naked GNCs, PEG-GNCs, and CD44-PEG-GNCs (3 nM) sharply increased from 30 to 53 °C and then slowly rose to 57–58 °C by 16 min. All of the tested GNCs could be heated, and no significant differences were found (*P* > 0.05). In contrast, a temperature rise was not obvious in the control group (Fig. 4).

### In Vitro Photothermal Therapy Effects

**Fig. 5:**
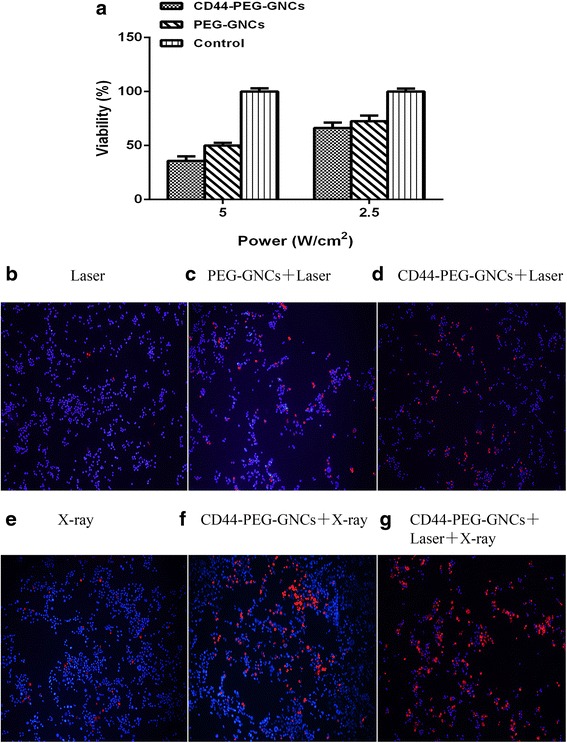
Different treatments of 4T1 cells. **a** 4T1 cells incubated with PEG-GNCs or CD44-PEG-GNCs for 24 h, then exposed to an 808-nm NIR laser (5 W/cm^2^, 2.5 W/cm^2^), measured by the CCK-8 assay. **b**–**g** Fluorescent images of 4T1 cells stained with a Hoechst/PI double staining kit. There were more dead cells in the CD44-PEG-GNCs group than in the control; the co-treatment group exhibited the greatest number of dead cells. These experiments were repeated 3 times, *P* < 0.05

4T1 cells were incubated with PEG-GNCs or CD44-PEG-GNCs for 24 h and then irradiated with an NIR laser (808 nm) at a power density of either 5 W/cm^2^ or 2.5 W/cm^2^ for 5 min. Cell viability was measured using a CCK-8 assay. As shown in Fig. 5a, the group incubated with CD44-PEG-GNCs and irradiated by the NIR laser (5 W/cm^2^) exhibited a much stronger growth inhibition than the other groups (*P* < 0.05). The cell viability of the CD44-PEG-GNC group was 38.86 %, while that of the PEG-GNC group was 50 % (*P* < 0.05). We further irradiated cells with a 2.5 W/cm^2^ NIR laser, and as the figure shows, the cells in the CD44-PEG-GNC group maintained 66.3 % viability, while the PEG-GNC group showed a viability of 72.6 % (*P* < 0.05). Thus, the cell viability rapidly decreased as the laser power increased. To specifically analyze the photothermal therapy effects, we applied double Hoechst/PI staining. Hoechst, a membrane-permeable dye, stains living cells blue, whereas PI stains cells that have lost membrane integrity red. Therefore, red cells represent dead cells, including necrotic or apoptotic cells, while blue cells represent living or early apoptotic cells. Fluorescent images showed that the control group, which was treated with the NIR laser alone, exhibited little change in cell viability. Meanwhile, the number of dead cells in the CD44-PEG-GNC group was significantly higher than those in the PEG-GNC group and the control group (Fig. 5b–d). In the above experiments, it was clear that GNCs can be used as photothermal conversion agents and that their efficiency is closely related to the power of NIR irradiation applied. To minimize damage to the surrounding normal tissue, we maintained the NIR power density at a low level of 2.5 W/cm^2^.

### In Vitro Enhanced Radiation Therapy Effects

**Fig. 6:**
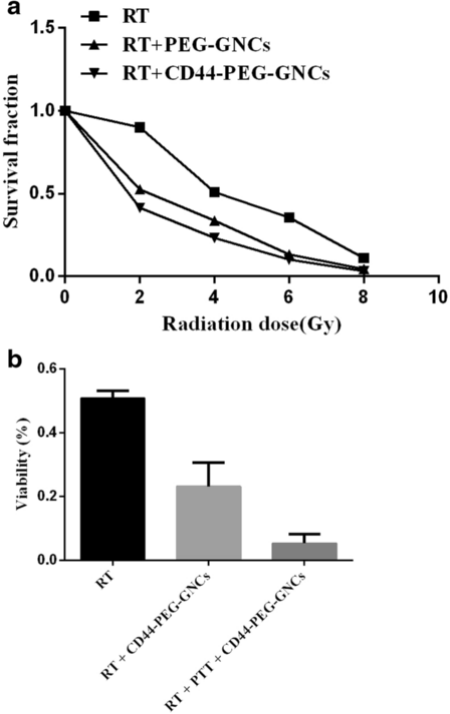
Analysis of the clonogenic survival assay results. **a** 4T1 cells incubated with PEG-GNCs or CD44-PEG-GNCs (0 or 3 nmol/l) for 24 h, then exposed to X-ray radiation. **b** 4T1 cells incubated with CD44-PEG-GNCs for 24 h, then exposed to NIR and X-ray radiation. These experiments were repeated 3 times, *P* < 0.05

To demonstrate that the GNCs can enhance radiosensitivity, we carried out a clonogenic survival assay. 4T1 cells were incubated with PEG-GNCs or CD44-PEG-GNCs for 24 h and then exposed to 2, 4, 6, or 8 Gy of 6-MV X-ray radiation. Figure 6a shows the inhibition rate of 4T1 cells incubated with PEG-GNCs or CD44-PEG-GNCs under different doses of X-ray irradiation. As indicated in this figure, the inhibition rate of the CD44-PEG-GNC group was significantly higher than that of the PEG-GNC group and the control group for the same dose of X-ray irradiation (*P* < 0.05). For example, when the X-ray dose was 2 Gy, the inhibition rate of the control group (irradiation alone) was 9.9 % for 4T1 cells, while the cell inhibition rates for the PEG-GNC and CD44-PEG-GNC groups increased to 47.468 and 58.6 %, respectively. In the double Hoechst/PI staining experiment, for the cells incubated with CD44-PEG-GNCs and subjected to X-ray (4-Gy) irradiation, the fluorescent images indicated a greater number of dead cells in comparison to the control group (Fig. 5e, f). Moreover, as the X-ray dose was increased, the inhibition rate of the 4T1 cells increased significantly. For example, the cell inhibition rates for control groups irradiated with X-ray doses of 2, 4, 6, and 8 Gy were 9.9, 49.12, 64.4, and 89.068 % (*P* < 0.05), respectively. These results indicate that the GNCs can be used as radiosensitizers [47].

### Apoptosis Detection

**Fig. 7:**
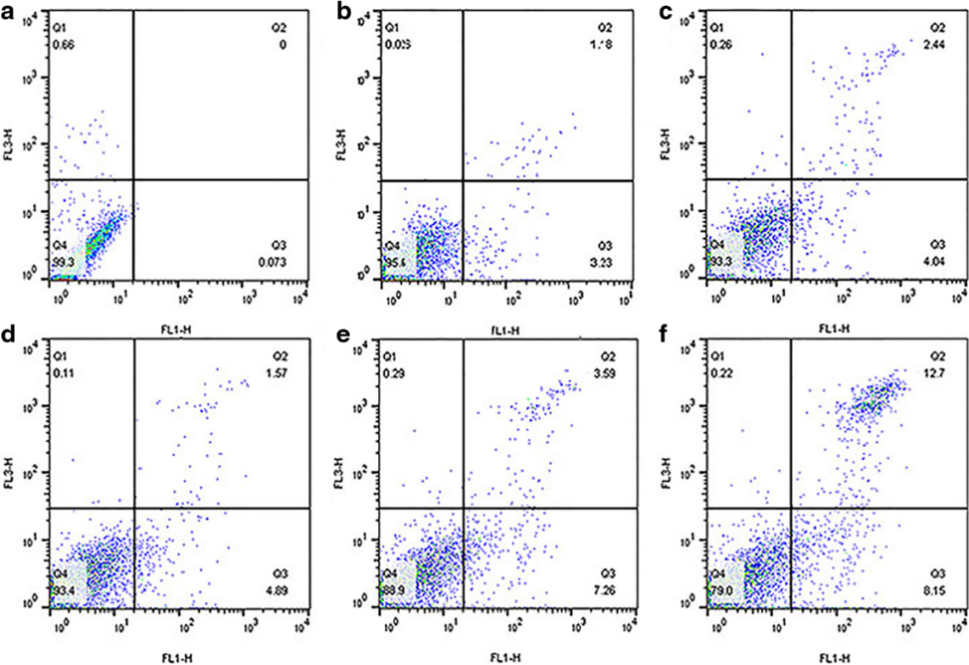
Analysis of apoptosis in 4T1 cells. Co-treatment led to a significant increase in apoptosis. **a** Control, **b** NIR light irradiation alone, **c** immuno GNCs + NIR, **d** X-ray irradiation alone, **e** immuno GNCs + X-ray, **f** immuno GNCs + NIR + X-ray

Cell apoptosis plays an important role in tumor cell growth; thus, we further assessed whether CD44-PEG-GNCs combined with NIR laser radiation and X-ray radiation can induce more apoptosis. 4T1 cells were incubated with 3 nM CD44-PEG-GNCs for 24 h and then exposed to NIR and/or X-ray irradiation for 5 min. Flow cytometry was employed to determine the extent of 4T1 cell apoptosis. Early- and late-stage apoptotic cells were primarily distributed in the lower and upper right quadrants, respectively, and dead cells are shown in the upper left quadrants. The apoptotic rate was calculated from the sums of the proportions of cells in the lower and upper right quadrants. As shown in Fig. 7, the combination of CD44-PEG-GNCs and NIR or X-ray irradiation induced more apoptosis (6.48 and 10.85 %, respectively) than NIR or X-ray irradiation alone (4.41 and 6.46 %, respectively) (*P* < 0.05). These data indicate that the use of combined treatment can increase cell apoptosis.

### Synergy Between Hyperthermia and Radiotherapy In Vitro

To further investigate the antitumor effect of the combination treatment of GNCs, PTT, and RT, double Hoechst/PI staining, a clonogenic survival assay, and apoptosis detection were employed. After 4T1 cells are irradiated by NIR light, X-ray irradiation should follow within 1 h. Double Hoechst/PI staining showed that the co-treatment of CD44-PEG-GNCs, PTT, and RT induced significantly more cell death than any other treatment (Fig. 5g). Moreover, the apoptosis rate and inhibition rate of the co-treatment group were 20.85 % (Fig. 7f) and 96 % (Fig. 6b), respectively, which are significantly higher than those of any other groups (*P* < 0.05). These data indicate that a combination strategy can increase the rate of cell apoptosis and inhibit cell proliferation.

### Hyperthermia and Radiotherapy in Mice

**Fig. 8:**
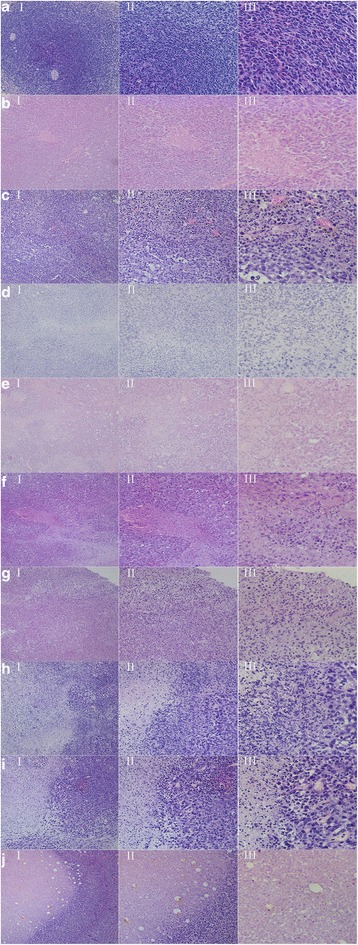
H&E staining of tumor tissues after different treatments. The sequence from **A** to **J** is as follows: the control group; PBS i.v. injection + PTT; PBS i.v. injection + PTT + RT; PEG-GNC i.v. injection + RT; CD44-PEG-GNC i.v. injection + PTT; CD44-PEG-GNC i.v. injection + RT; CD44-PEG-GNC i.v. injection + PTT + RT; GNC i.t. injection + RT; GNC i.t. injection + PTT; GNC i.t. injection + PTT + RT. Sections were observed under 10× (*I*), 20× (*II*), and 40× (*III*) magnifications

To assess the antitumor effects of GNCs, we employed H&E staining to observe histopathologic changes in tumors. H&E staining showed common tumor tissue with large, deeply stained, and tightly packed nuclei ((Fig. 8a) I-8(A) III). Likewise, in the PBS group, with either NIR or X-ray treatment, there was no significant cell damage, few tumor cells were necrotic, and the tumor cells were arranged in a slightly loose manner ((Fig. 8b) I-8(B) III). In the PBS combined with PTT + RT group, a small amount of cell shrinkage and karyorrhexis was observed ((Fig. 8c) I-8(C) III). Compared with the PBS group, the number of necrotized cells was higher in the group treated with PTT or RT combined with the i.v. injection of PEG-GNCs ((Fig. 8d) I-8(D) III). Moreover, karyorrhexis, karyolysis, and inflammatory cell infiltration were observed in the tumor tissues. H&E staining confirmed that the combination of CD44-PEG-GNCs administered by i.v. injection followed by PTT or RT led to significant necrosis of more cells, corruption of the extracellular matrix, and more cytoplasmic acidophilia compared to the PBS group and the untreated group ((Fig. 8e) I-8(E) III and 8(F) I-8(F) III). Moreover, the tumors in the combined PTT + RT group exhibited more karyorrhexis, karyolysis, and inflammatory cell infiltration ((Fig. 8g) I-8(G) III). To compare the effect of different injection therapies, we also conducted an i.t. injection. In the group treated with PTT or RT combined with i.t. injection of GNCs, the tumor cells exhibited focal coagulative necrosis. The cells lost their normal structure with large amounts of inflammatory cell infiltration ((Fig. 8h) I-8(H) III and 8(I) I-8(I) III). Moreover, following i.t. injection of GNCs combined with PTT and RT, almost all of the tumor tissue showed coagulative necrosis. In the necrotic area, the tumor tissue showed homogeneous red staining with a loss of cell structure. In the boundary of the necrotic area, a large amount of inflammatory cell infiltration was found ((Fig. 8j) I-8(J) III). These results confirm that the PTT and RT used in this study did not show obvious treatment effects in the absence of GNCs and that the CD44-PEG-GNCs could induce significantly more tumor tissue necrosis.

## Discussion

Due to their excellent biocompatibility and biological functions, gold nanoparticles have been widely explored in translational research. Among different gold nanostructures, GNCs are promising nanoparticles for cancer theranostics. To enhance the stability of GNC suspensions in vivo, hydrophilic polymer ligands, such as PEG, have been widely used in biological nanotechnology. PEGylated GNCs not only have increased hydrophilicity but also have decreased recognition and uptake by the reticuloendothelial system (RES) [25]. As such, PEGylated GNCs have an increased circulatory half-life, which is a very important parameter for the passive targeting of nanoparticles. In the current study, we used bi-functional thiol-containing PEG, which is easy to conjugate to both GNCs and antibodies. To meet the nutritional requirements needed for accelerated growth and division, solid tumors possess leaky vasculature and diminished lymphatic drainage, which also contribute to the retention of GNCs at the tumor site. This phenomenon is known as the enhanced permeability and retention (EPR) effect, which is an important basis for passive targeting [25]. However, Devika et al. found that, in the intracellular environment, AuNPs contribute better radiosensitization effects than those of extracellular particles [52]. As RME is the main way that cells uptake particles, in the current study, we constructed CD44-PEG-GNCs. Many studies have reported that CD44 receptors are over-expressed on the cell membrane of many aggressive tumor cell types [55], such as in breast cancer [56], prostate cancer [57], pancreatic carcinoma [58, 59], ovarian cancer [60], and gastric carcinoma [61]. In recent studies, CD44 has been recognized as a pleiotropic molecule related to tumor metastasis and aggressive behavior [62] and has become an attractive target for antitumor theranostics. Our immunohistochemical results showed that a large number of CD44 receptors were expressed on the 4T1 cell membrane, while few were observed in the cytoplasm, indicating that CD44-PEG-GNCs can be specifically delivered to 4T1 cells. ICP-MS was used to perform quantitative comparative analysis of the numbers of CD44-PEG-GNCs, PEG-GNCs, and GNCs aggregated within 4T1 cells, and TEM was used to visualize the GNC distribution in the cells. GNC size is another factor related to targeted cellular uptake. Chithrani et al. compared AuNPs with diameters of 14, 30, 74, and 100 nm and found that the 50-nm-diameter AuNPs had the most efficient cellular uptake [63]. In the current study, the mean diameter of our GNCs was 58.14 ± 4 nm. Furthermore, Zhang et al. reported that AuNP cytotoxicity is also associated with particle size. After incubating HeLa cells with variously sized particles at a fixed concentration (0.25 mM) for 24 h, the cell viability was 90 % for 46.6-nm-diameter PEGylated AuNPs compared to 50 and 60 % for 4.8-nm and 27.3-nm-diameter particles, respectively, demonstrating that the larger particles had the best biocompatibility [64].

Hyperthermia, such as radiofrequency ablation and microwave hyperthermia, is an effective treatment that can change the microenvironment and signal transduction of a tumor [19, 20], as when a temperature of 40 °C is reached, irreversible damage due to protein denaturation and DNA double-strand repair deficiency will occur. However, hyperthermia is limited in clinical therapy because it is difficult to target this approach to specific tissues [19, 20]. Therefore, specific targeting materials must be used when applying hyperthermia in the clinic. Previous research has shown that gold nanoparticles are excellent photothermal transducers that can absorb NIR and efficiently convert it to heat [7]. Due to the biological transparency window (700–900 nm), NIR light is well-suited for photothermal therapy with excellent light penetration and minimal damage to normal tissues [7, 16, 47]. Thus, our immuno GNCs can achieve tumor-specific heating with little damage to normal tissue. However, using a temperature that is too high would inevitably cause adverse side effects; therefore, we used temperatures within the range of 40–43 °C. In our study, the results of in vitro PTT indicate that the combination of GNCs with the local application of NIR irradiation influences 4T1 cell viability. The efficiency of CD44-PEG-GNCs with NIR irradiation was evident with a 61.14 % mortality rate of 4T1 cells, while GNCs or NIR irradiation alone had no obvious effects. We further investigated the effect of laser power density on cell activity and found that the rate of cell death increased significantly with increasing power. This positive relationship indicates the importance of selecting the appropriate power to minimize damage to nearby normal cells. To better visualize the effects produced by PTT, we used double Hoechst/PI dye staining. Living cells were stained blue with Hoechst, while dead cells were stained red with PI. Fluorescence imaging showed that the group treated with CD44-PEG-GNCs combined with PTT had more dead cells than the control groups.

Due to their high atomic number, the GNCs can deposit significantly more X-ray irradiation in the tumor cells when they accumulate [19, 47, 65]. This phenomenon may be applied to resolve the problem of radiation resistance and side effects in clinical therapy. The results of a clonogenic survival assay suggest that for a given X-ray irradiation dose, the inhibition rate of the CD44-PEG-GNC group was significantly higher than that of the PEG-GNC group and the control group. The effect of enhanced radiosensitivity was also investigated by double Hoechst/PI dye staining and an apoptosis assay.

Several studies have shown that PTT is one of the most effective radiosensitizers [19]. Although the mechanisms of thermal enhancement in RT are not yet fully understood, the inability to repair double-stranded breaks caused by RT and alterations in heat shock proteins must play a pivotal role [7, 48]. In the current study, we kept the temperature within the range of 40–43 °C; in this range, PTT can reduce tumor hypoxia by increasing blood perfusion [66]. Hypoxia is known to induce tumor aggressiveness and resistance to RT [66, 67]. We also used double Hoechst/PI staining to validate that the combination therapy is much more efficient than PTT or RT alone. Furthermore, we employed an apoptosis assay and a clonogenic survival assay to quantitatively compare the effects of the different therapeutic strategies. Apoptosis plays a vital role in regulating cell death and is very important in cancer therapy [11, 50]. One significant characteristic of tumor development is the evasion of apoptosis [11]. In our study, a dramatic increase of apoptosis in 4T1 cells due to the use of a combination of CD44-PEG-GNCs, PTT, and RT was observed by flow cytometry. Furthermore, a clonogenic survival assay can be used to reflect the proliferation and invasion of tumor cells and to evaluate the sensitivity of tumor cells to antitumor factors. The group treated with a combination of CD44-PEG-GNCs, PTT, and RT exhibited a remarkable inhibition rate (96 %) compared to the group treated with RT combined with CD44-PEG-GNCs (76.8 %) and the group treated with RT alone (49.12 %). In summary, our in vitro studies indicate that the co-treatment of GNCs, PTT, and RT may be a promising approach for cancer therapy.

Histological analyses of tumor tissues stained with H&E were performed to evaluate the cellular effects of treatments when combined with immuno GNCs. Our results indicate that the combination of GNCs administered by either intratumoral (i.t.) or intravenous (i.v.) injection followed by PTT and RT treatment caused significantly greater cell necrosis and corruption of the cytoplasm and extracellular matrix in comparison to PBS with PTT or RT. Jaesook et al. suggested that PTT may increase blood flow in tumors and reduce the hypoxic region, which sensitizes the tumor to radiotherapy [47], thus indicating that PTT is an excellent radiosensitizer. Moreover, Hainfeld et al. reported that GNCs can distribute heat to surrounding cells by conduction, convection, and radiative transfer, which expands the region of the tumor being heated. Although the therapeutic efficacy of PTT therapy alone is poor, this approach is effective when combined with radiation [19]. Our studies also compared the results for intratumoral injection and intravenous injection. As Fig. 8j shows for a tumor treated with i.t. injection plus PTT and RT, almost all of the tissues were necrotized, cytoplasmic acidophilia occurred, and the extracellular matrix was corrupted. However, it is difficult for GNCs to spread uniformly in a tumor, and GNCs can also easily escape from a tumor. Moreover, the tumor boundary is not clear, in general, and the actual size is generally much larger than what can be seen. For this reason, we could not determine whether the tumor cells were completely killed by the treatment with the i.t. injection. In contrast, if the amount of GNCs is sufficient, the intravenous delivery of GNCs can cover the growing edge. Hainfeld et al. suggested that internal tumor cells may not be able to survive if the growing edge of the tumor is completely destroyed [19]. In our study, for the tumor treated with i.v. injection plus PTT and RT, most of the cells exhibited pyknosis and karyolysis, and the extracellular matrices were corrupted (Fig. 8g). However, there are benefits to i.t. injection. This type of injection can ensure that high amounts of GNCs are present in the tumor site with low toxicity to the body, which is difficult to achieve with i.v. injection.

## Conclusions

In our study, we synthesized CD44-PEG-GNCs that exhibit promising effects for cancer therapy. We showed that the efficiency of the immuno GNC cellular uptake was significantly greater compared to PEGylated GNCs. Furthermore, the combination treatment of CD44-PEG-GNCs, PTT, and RT was evaluated in vitro and in vivo. The in vitro studies illustrated that the novel integrated therapy was the most effective in 4T1 cells. In addition, we compared the effect of different treatments on tumor tissues and found that the use of intravenous injection plus PTT and RT can successfully kill tumor cells with little damage to the normal tissue. We envision that these GNCs could be applied for human cancer patients to overcome resistance to radiation therapy with few side effects. Future work should address the use of GNCs in an orthotopic tumor model to provide a more accurate reference for clinically relevant evaluations.
